# A refined approach to compute nanodosimetric quantities for proton and ion radiotherapy treatment planning

**DOI:** 10.1088/1361-6560/ae2230

**Published:** 2025-11-28

**Authors:** Ramon Ortiz, José Ramos-Méndez, Naoki D-Kondo, Bruce Faddegon

**Affiliations:** Department of Radiation Oncology, University of California San Francisco, San Francisco, CA, United States of America

**Keywords:** nanodosimetry, cluster dose, ICSD, ionization parameter, particle therapy

## Abstract

*Objective*. To present a refined approach to calculate a database of nanodosimetric quantities and efficiently and accurately compute voxel-averaged ionization detail (ID) quantities for particle radiotherapy treatment planning (RTP) applications. *Approach*. Monte Carlo track structure was employed to compute nanodosimetric quantities at nanoscale. The new approach includes the extension of particle energy ranges to encompass clinically relevant values, consideration of isotopes, optimally spaced energies, and consistent treatment of secondary particles at nano and macroscale through application of common production cutoffs to avoid double-counting/omission of ionizations. At macroscale using Monte Carlo condensed history simulations, we devised a scoring approach to account for ID quantity changes across Monte Carlo steps with each step divided into substeps chosen to be consistent with database energies. The database calculated with the refined approach was compared to our previously published approach and we studied its impact on the identification of preferred ID quantities for their closest association with cell survival in three biological datasets. *Results*. The optimized energy binning reduced interpolation errors to below 1%. Across different isotopes, the ID quantities differed on average by 2% relative to that of the corresponding stable ion, resulting in an error of less than 0.3% when using a single ID value per atomic number in macroscopic calculations. Our refined approach revealed differences from our previous method resulting in increases of up to 20% and 10% in cluster dose SOBP’s for carbon and oxygen ions, three-fold for protons. In addition, application of the refined approach altered the selection of the preferred ID quantity in one of the three datasets studied, highlighting that these refinements can influence biologically informed choices of nanodosimetric quantities. *Significance*. This work provides improved methodology for integration of nanodosimetric parameters into RTP by bridging the gap between nanoscale ionization processes and macroscopic cluster dose calculations.

## 1. Introduction

Currently, ion radiotherapy treatment planning (RTP) relies on model-based RBEs, yet significant discrepancies exist between models and across treatment centers (Fossati *et al* 2018). Recent work has highlighted linear energy transfer (LET) as a surrogate quantity (McIntyre *et al* 2023), supporting its visualization and use in treatment plan optimization (Harrison *et al* 2024), that can be validated microscopically (Harrison *et al* 2024). However, LET remains an indirect descriptor, and biological effects can differ significantly for particles with the same LET. Recent radiobiological insights emphasize the importance of ionization clustering and the induction of complex DNA lesions (Hagiwara *et al* 2019, Chatzipapas *et al* 2020, Nikitaki *et al* 2020, Falk and Hausmann 2021, Wozny and Rodriguez-Lafrasse 2023), which more directly describe cell fate than LET or RBE.

Nanodosimetry is the study of the spatial distribution of interaction events, primarily ionizations, of radiation in matter. When the nanoscopic volumes used to score ionization events resemble biological volumes such as short segments of DNA, certain nanodosimetric quantities correlate with key biological effects in cancer therapy, including cell inactivation, DNA double-strand break (DSB) cross-sections, and residual DNA damage (Blakely 1992, Nettelbeck and Rabus 2011, Conte *et al* 2012, 2017, 2018, 2023, Rabus *et al* 2020, Faddegon *et al* 2023, Mietelska *et al* 2024). This highlights the relevance of nanodosimetric quantities in understanding the biological effect of radiation.

Several studies have proposed the integration of nanodosimetric quantities into RTP as a strategy to improve treatment through biologically optimized ion radiotherapy (Villegas *et al* 2016, Ramos-Méndez *et al* 2018, Burigo *et al* 2019, Dai *et al* 2020, Rucinski *et al* 2021, Faddegon *et al* 2023). This integration poses the challenge of bridging two disparate spatial scales: the nanometer scale for consideration of the detailed spatial deposition of energy and the resulting pattern of ionization along a particle track, or ionization detail (ID), and the millimeter scale for consideration of RTP at the patient level. In a previous work (Faddegon *et al* 2023), we established an ID-based mathematical formalism to provide the practical means to incorporate nanodosimetric quantities in RTP. This approach builds upon the generalized definition of ionization cluster size *ν* and its frequency distribution *f(ν)*. The frequency ionization cluster size distribution fICSD, or *f(ν)*, can be collapsed into a scalar (or vector) ionization parameter *I*_p_. These quantities are defined per unit track length of a specific particle with a specific energy. To accommodate practical considerations in RTP, such as optimizing the source fluence of individual pencil beams (Ortiz and Faddegon 2024), two macroscopic quantities were introduced: the voxel-averaged *I*_p_ (averaged over all charged particle track segments within a millimeter-sized voxel such as used in RTP) and the generalized cluster dose *g^(Ip^^)^*. Cluster dose, the product of the charged particle fluence within a macroscopic voxel and the voxel-averaged *I*_p_, is a generalization of the number of clusters per unit volume. Previous studies already showed a close association of certain *I*_p_ and cluster dose with clonogenic cell survival. For the preferred definition of *I*_p_, which is the *I*_p_ that leads to the same cell survival at constant fluence, for the purposes of ID-based RTP, the same survival ideally has the same cluster dose, independent of particle type, energy and fluence. A preliminary evaluation of cell survival data for two cell lines, that cover a wide range of particle type and energy, found preferred *I*_p_ definitions for the set {*F*_k_*, k =*1*,…*10}, the number of clusters of *k* or more ionizations in volumes resembling short segments of DNA, to be *F_5_* and *F_7_* in aerobic and hypoxic conditions, respectively (Faddegon *et al* 2023). Remarkably, these definitions resulted in the same survival for the same cluster dose for human alveolar adenocarcinoma A549 and human kidney T-1 cell lines, a desirable property for ID-based RTP (Ortiz *et al* 2025).

Due to the high level of spatial detail required for computing nanodosimetric quantities, performing these calculations in real time at the patient level is not feasible within practical time constraints. To address this, a two-step approach has been proposed for integrating nanodosimetric quantities into patient-level calculations. In the first step, nanodosimetric quantities are precomputed for a broad range of particle types and energies using Monte Carlo track structure (MCTS) simulations. These simulations provide the necessary level of detail by computing nanodosimetric quantities within nanometric volumes. MCTS calculation at nanoscale provides a pre-calculated database containing fICSD and/or *I*_p_. In the second step, the database computed at nanoscale serves as a look-up table in Monte Carlo condensed history (MCCH) simulations or pencil-beam algorithms, enabling efficient computation of voxel-averaged *I*_p_ and cluster dose distributions at the patient level. Specifically, when a charged particle undergoes an interaction in MCCH, the particle type and energy are used to retrieve the corresponding *I*_p_ from the database pre-calculated at nanoscale, and the voxel-averaged *I*_p_ is computed as the track-weighted sum of the *I*_p_ of all charged particles interacting within the voxel divided by the sum of the track lengths. From the voxel-averaged *I*_p_ distribution at patient level, cluster dose is computed per voxel as described in section 2.1. While preliminary databases have been computed in previous studies to support the demonstration of the feasibility and potential of ID-based RTP formalism (Ramos-Méndez *et al* 2018, Faddegon *et al* 2023), further refinements are needed to improve accuracy and ensure a consistent, compatible integration of nanoscopic and macroscopic scales.

The purpose of this work was to construct a comprehensive fICSD database calculated at an optimal set of energies for all particle types that contribute significantly to ID, accounting for particle track ends without double counting ionization events in the transition from nanoscopic to macroscopic scales. In order to show the impact of the newly calculated database on ID-based RTP, we devised an approach for computing nanodosimetric parameters for RTP at the macroscale, ensuring compatibility with the database structure.

## 2. Materials and methods

### 2.1. General description of the ID formalism

This section provides a concise introduction to the nanodosimetric quantities in the ID-based RTP formalism relevant to this work. A detailed description can be found elsewhere (Faddegon *et al* 2023).

Ionization cluster size, *ν*, is the number of ionizations produced in nanometric sampling volumes (see section 2.2). A large number of sampling volumes are enclosed in a larger simulation volume. A charged particle traversing the simulation volume may produce ionizations in some of the enclosed sampling volumes, resulting in clusters of different sizes. The accumulation of cluster sizes from the traversal of many particles results in the fICSD (i.e. *f(ν)*) for that particular particle type and energy. Since *f(ν)* is defined as an absolute frequency distribution per unit track length of the particle, it is independent of particle fluence. Ionization parameters, *I*_p_, are calculated by applying an operator *G*_p_ to *f(ν)*:

\begin{equation*}{{\text{I}}_{\text{p}}}{\text{ = }}{G_{\text{p}}}{{\left[ {\text{f}}\left(\nu \right)\right]}}.\end{equation*}

A relevant example of *I*_p_ definition for its association with biological endpoints (Faddegon *et al* 2023) is *F*_k_, defined as the number of clusters of *k* or more ionizations:

\begin{equation*}{{\text{F}}_{\text{k}}}{\text{ = }}\mathop \sum \limits_{{{\nu = {\text{k}}}}}^{{{{\nu }}_{{\text{max}}}}} {{ \text{f}\left(\nu \right)}}.\end{equation*}

The quantities *f(ν)* and *I*_p_ are calculated for a set of particle classes ${{\mathrm{c}} \in \mathcal{C}_{\text{j}}}$, i.e. particle types and energies. This constitutes the database of nanodosimetric quantities. Section 2.2 describes the methodology proposed in this work to compute the ID database.

This database can be used in macroscale simulations for the calculation of voxel-averaged *I*_p_ and cluster dose in macroscopic volumes used in RTP voxels. In MCCH simulations, the track of each particle in a voxel is subdivided into shorter track segments, with particle type and energy for each segment equated to a single particle class. Voxel-averaged (macroscopic) *I*_p_ (or fICSD) is calculated by summing up the product of pre-calculated database *I*_p_ (or fICSD) with the summed length of the track segments of particles of each class interacting in a voxel:

\begin{equation*}{\text{I}}_{\text{p}}^{{{{\varphi }}_{\text{j}}}} = \frac{{\sum\nolimits_{{\text{c}} \in {{\mathcal{C}}_{\text{j}}}} {{\text{t}}_{\text{j}}^{\text{c}}{\text{I}}_{\text{p}}^{\text{c}}} }}{{\sum\nolimits_{{\text{c}} \in {{\mathcal{C}}_{\text{j}}}} {{\text{t}}_{\text{j}}^{\text{c}}} }},\end{equation*}

where ${\text{t}}_{\text{j}}^{\text{c}}$ is the track length of particle class $c$ in the set of charged particles interacting in the voxel *j*. Note that fICSD, ${\text{I}}_{\text{p}}^{\text{c}}$ and ${\text{I}}_{\text{p}}^{{{{\varphi }}_{\text{j}}}}$ have dimensions of length^−1^ and are independent of fluence. Cluster dose, ${\text{g}}_{\text{j}}^{{\text{(}}{{\text{I}}_{\text{p}}}{\text{)}}}$, is computed as the product of the fluence of charged particles, ${\phi _j}$, and the mean mass voxel-averaged ${I_{\text{p}}}$, i.e. $I_{\text{p}}^{{\mathcal{C}_j}}/{\rho _o}$:

\begin{equation*}{\text{g}}_{\text{j}}^{{\text{(}}{{\text{I}}_{\text{p}}}{\text{)}}}: = {\phi _{\text{j}}}{\text{ I}}_{\text{p}}^{{\mathcal{C}_{\text{j}}}}{\text{/}}{{{\rho }}_{\text{0}}},\end{equation*}

where ${\rho _o}$ is the density of the material used to compute the nanoscopic *f(ν)*. In the case of *F*_k_, cluster dose is the total number of ionization clusters of *k* or more ionizations produced per unit mass by the set of particles interacting in a given voxel within the patient, i.e. the unit of cluster dose is the reciprocal of mass, for instance 1 pg^−1^.

### 2.2. Calculation of database at nanoscale

The following subsections describe the methodology proposed in this work to compute the database of ID quantities. TOPAS-nBio (Schuemann *et al* 2018), the track structure extension of the TOPAS toolkit (Perl *et al* 2012, Faddegon *et al* 2020) was used for the computation of the database. A developer version of TOPAS-nBio v3.0, built on TOPAS version OpenTOPAS v.4.0 (Geant4-11.1.3) downloaded from https://opentopas.github.io/, was employed. The TsEmDNAPhysics physics module, consisting of the Geant4 G4EmDNAPhysics_option2 module, and TsEmDNAChemistry chemistry module, were used with default options for all transportation parameters. For the thermalization of subexcited electrons and elastic scattering, the Meesungnoen model and ELSEPA model were used, respectively. Previous databases were calculated with TOPAS-nBio (v.2.0), built on TOPAS version 3.9 (Geant4-10.7). The Monte Carlo codes employed in this work have been updated and extensively validated for radiation transport across different scales (Incerti *et al* 2010, Bernal *et al* 2015, McNamara *et al* 2017, Masilela *et al* 2025) and have demonstrated good agreement with experimental nanodosimetric measurements (Burigo *et al* 2016).

#### 2.2.1. Geometry

**Figure 1.**
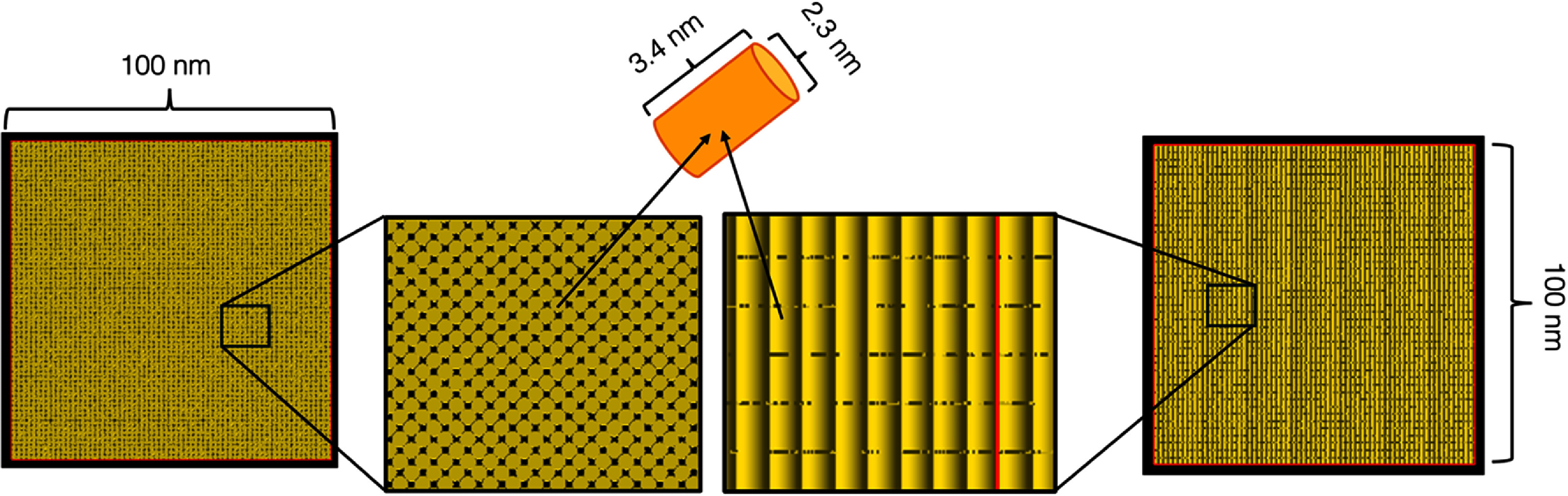
Schematic representation of simulation and sampling volumes. The simulation volume is shown in red and the sampling volumes in yellow.

The geometry for computing the database of nanodosimetric quantities consists of a 100 × 100 × 100 nm^3^ cube of water as a simulation volume (TsBox component in TOPAS). A sphere or cylinder could not be used with the current TOPAS implementation of periodic boundary conditions (PBCs). Cylinders of 2.3 nm diameter and 3.4 nm long were used as sampling volumes, i.e. volumes where the ionization clusters were scored. Nanodosimetric quantities are known to vary with the choice of simulation volume geometry, e.g. larger sampling volumes increase the frequency of larger clusters. This sampling volume geometry was chosen to resemble the size and shape of a DNA segment of 10 base pairs, where single damages in DNA strands can form clustered DNA lesions (e.g. DSB) (Charlton *et al* 1989), leading to biological consequences of relevance in cancer therapy (e.g. cell death). The simulation volume was filled by the maximum number of sampling volumes (53621 cylinders; 76% of simulation volume) oriented along its *Z*-axis with no overlap, with adjacent rows of cylinders shifted by the radius of the cylinder (figure 1). This tight packing was done to improve the efficiency in ionization cluster scoring. The geometry used for previously calculated databases consisted of 1800 sampling volumes randomly distributed in a 30.4 nm diameter and 161 nm long cylindrical simulation volume (22% of simulation volume filled by sampling volume).

Both simulation and sampling volumes were made of water (*G4_WATER* material with mean excitation energy of 78.0 eV and unit density). This is in part due to water being the predominant material in a living cell and the limited availability of cross-sections of other relevant biological materials at the nanoscale. In the calculation of voxel-averaged *I*_p_ and cluster dose, the track lengths scale with the density of the material (tissue) in the volume compared to the density of the medium in the MCTS simulations used to calculate the database quantities for each particle class, accounting for the linear increase in these quantities with density (Faddegon *et al* 2023).

#### 2.2.2. Radiation source

The particle source was randomly dispersed within a sphere positioned at the center of the simulation volume. The source volume was made of vacuum and had a diameter of 34 nm, chosen to be ten times larger than the maximum size of the sampling volumes. No sampling volumes were included in the source volume to avoid geometrical overlaps, since in the current version of TOPAS, parallel volumes are not compatible with the pre-chemistry processes used in this work. Therefore, the source volume could not be defined as a parallel volume to prevent the overlap. Particles were generated isotropically at any point within this sphere. This configuration was designed to resemble the random position and orientation of short DNA segments relative to incoming particles in real scenarios and minimizes the influence of the orientation of sampling volumes described in section 2.2.1. The orientation of the cylinders showed no significant effect on relative fICSD compared with randomly oriented sampling volumes (data not shown) due to the use of an isotropic particle source, while the tight packing of sampling volumes aligned in the same direction improved simulation efficiency. Differences are expected when there exists a preferential particle direction, as discussed in Ramos-Méndez *et al* (2018). This source configuration allowed aligning the simulation volumes in a common direction to maximize the number of sampling volumes within the simulation region, enhancing the computational efficiency of the database calculation (see section 2.2.1). Each particle history (a primary particle and all its descendant secondary particles) was transported and scored independently, thus no inter-track effects were considered.

As detailed in section 2.1, the database is composed of nanodosimetric quantities computed for individual particle classes (i.e. particle types and energies). We included particle types of relevance in current proton and ion therapy applications and secondary charged particles that primary ions may create in the patient volume, i.e. electrons, protons, and ions from ^4^He to ^40^Ar. Fully stripped protons and ions were used. Only stable isotopes, i.e. those present in the periodic table, were considered. The change in database quantities among different isotopes was studied for a selected set of isotopes and energies to validate their exclusion from the database: 6-Li, 7-Be, 10-B, 13C, 15-N, 15-O, 18-F, 21-Ne, 22-Na, 25-Mg, 28-Al, 29-Si, 32-P, 33-S, 36-Cl, and 39-Ar at the lowest and highest energies presented in table 1. These isotopes were chosen for their higher abundance at the distal edge of a pristine Ar-ion Bragg peak (data not shown), as determined from TOPAS simulations.

**Table 1.** Range of energies included in the database for each particle type.

Particle type	*E*_min_	*E*_max_	Particle type	*E*_min_	*E*_max_
Electron	1 keV *	1 MeV ***	Ne	0.5 MeV u^−1^ **	600 MeV u^−1^ ****
Proton	100 keV *	230 MeV ****	Na	0.5 MeV u^−1^ **	615 MeV u^−1^ ****
He	0.5 MeV u^−1^ **	100 MeV u^−1^ ***	Mg	0.5 MeV u^−1^ **	675 MeV u^−1^ ****
Li	0.5 MeV u^−1^ **	255 MeV u^−1^ ****	Al	0.5 MeV u^−1^ **	700 MeV u^−1^ ****
Be	0.5 MeV u^−1^ **	305 MeV u^−1^ ****	Si	0.5 MeV u^−1^ **	760 MeV u^−1^ ****
B	0.5 MeV u^−1^ **	355 MeV u^−1^ ****	P	0.5 MeV u^−1^ **	780 MeV u^−1^ ****
C	0.5 MeV u^−1^ **	425 MeV u^−1^ ****	S	0.5 MeV u^−1^ **	840 MeV u^−1^ ****
N	0.5 MeV u^−1^ **	470 MeV u^−1^ ****	Cl	0.5 MeV u^−1^ **	900 MeV u^−1^ ****
O	0.5 MeV u^−1^ **	510 MeV u^−1^ ****	Ar	0.5 MeV u^−1^ **	950 MeV u^−1^ ****
F	0.5 MeV u^−1^ **	530 MeV u^−1^ ****	—	—	—

* The default energy cutoff in MCCH simulations, i.e. *G4EmStandardPhysics_option4* physics module.

** Lowest valid energy in Geant4-DNA ionization models.

*** High valid energy in Geant4-DNA ionization models.

**** Highest clinically relevant energy, i.e. range ∼30 cm in water of monoenergetic beam.

The range of source energies for each particle was determined as follows:

1. The energy range was limited to those energies for which ionization models are valid in Geant4-DNA code.
2. The energy range included the full range of clinically relevant energies.
3. The default energy cutoff for the transport of particles in TOPAS MCCH simulations was considered. Note that in MCCH simulations, particles with a lower energy that the transport cutoff are not tracked, and the remaining kinetic energy is deposited locally. Energies lower than default cutoff were not computed since they would not be used in macroscopic scale simulations.

The ionizations produced by these energies were included in the database calculations as described in section 2.2.3. The energy ranges for each particle type are summarized in table 1. Note that for ions heavier than helium with energies below 1 MeV u^−1^, the currently available cross sections in Geant4-DNA include only ionization, excluding charge exchange and excitation processes. The database for this energy range may need to be updated in the future when revised cross sections become available to enable a more comprehensive representation of their physical interactions at the nanoscale. Importantly, these energy ranges extend the lower limits the energies for all particle types in previous databases where the lowest electron, proton and ion energies were 10 keV, 0.5 MeV, and 1 MeV u^−1^ respectively (Faddegon *et al* 2023). In previous approaches, these lower limits were set to the minimum energy required for the particle to traverse a 30.4 nm diameter simulation volume or the minimum energy that could be simulated in Geant4-DNA, whichever was greater. The mean chord length of the simulation volume (average length of straight-line paths through the volume) was used as the average primary particle track length through the volume and served to define the nanoscopic fICSD and *I*_p_ per unit track length in the database, as described in section 2.1. As detailed in section 2.2.3, in the proposed approach, the track length of each primary particle through the simulation volume is individually computed, accounting for potential scattering (relevant for electrons), and allowing particles to stop in the simulation volume, to extend the database to lower energies. Only the track length within the simulation volume, excluding the source volume (composed of vacuum), was considered, since this is the portion of the track that contributes to the fICSD. The mean track length of particles within the source volume was 12.75 nm.

Within these ranges, nanodosimetric quantities were computed for a set of initial particle energies. The energy loss of primary particles within the simulation volume was limited as described in section 2.2.3. The energy spacing between the database energies was determined to obtain a maximum variation of 5% in *F_5_* to *F_7_* ionization parameters. That is, the minimum change in energy that results in 5% variation steps for *F_5_* to *F_7_* is set as the energy step. The range of *I*_p_ from *F_5_* to *F_7_* were selected as representative definitions of *I*_p_ due to their close association with cell survival as previously noted for human alveolar adenocarcinoma A549 and human kidney T-1 cell lines. Note that in MCCH calculations, if a particle energy at the midpoint of a given step is not explicitly included in the database, the *I*_p_ is linearly interpolated. For the energy spacing proposed, the value of *F*_k_ for any intermediate energy is obtained by linear interpolation within 1% difference from the *F*_k_ value computed for that exact energy.

The number of simulated initial histories was determined per particle class (i.e. particle type and energy) to obtain a statistical uncertainty in the computation of in F_5_ to F_7_ ⩽ 1%. The mean number of original histories was (to two significant figures) 5.1 × 10^5^ for electrons, 1.3 × 10^8^ for protons, 7.0 × 10^6^ for He, 1.3 × 10^6^ for Li, 8.8 × 10^5^ for Be, 6.1 × 10^5^ for B, 3.4 × 10^5^ for C, 2.6 × 10^5^ for N, 1.9 × 10^5^ for O, 1.3 × 10^5^ for F, 1.2 × 10^5^ for Ne, 8.1 × 10^4^ for Na, 7.0 × 10^4^ for Mg, 5.2 × 10^4^ for Al, 4.1 × 10^4^ for Si, 3.5 × 10^4^ for P, 3.3 × 10^4^ for S, 3.0 × 10^4^ for Cl, and 2.5 × 10^4^ for Ar. The statistical uncertainty was calculated as the standard error of ten independent simulations.

#### 2.2.3. Contributions of primary and secondary particles

Nanodosimetric quantities in the database were computed per initial primary particle and per track length of the primary particle in the simulation volume. This computation includes the ionizations produced by primary particles and their secondary particles.

The point of ionization was defined as the position of the ionized species (i.e. ionized water molecules, H_2_O^+^) at the pre-chemical stage of the interaction, as in previous approaches (Faddegon *et al* 2023).

Primary particles were terminated (i.e. no longer tracked) based on their energy loss within the simulation volume. Specifically, a primary particle was killed when its energy loss exceeded 1% of the difference between its initial energy and the next lower adjacent energy in the database. For example, if a particle started with an energy of 15 MeV and the next lowest energy point in the database was 10 MeV, the maximum allowed energy loss was 0.05 MeV. The rationale behind this approach was to ensure that nanodosimetric quantities were computed for a set of nearly monoenergetic primary particles. This allowed for accurate interpolation of fICSD and *I*_p_ values for intermediate energies while preventing double counting of ionization clusters. Without this condition, overlapping energy ranges could lead to redundant ionization scoring. Additionally, restricting calculations to nearly monoenergetic primary particles and using interpolation for intermediate energies significantly reduces database calculation time. Allowing particles to be transported down to the next energy bin would require a substantially larger simulation volume and an increased number of sampling volumes, dramatically extending simulation times, particularly for high-energy particles.

The contribution of secondary electrons, positrons and protons to the fICSD calculations was considered based on a production threshold applicable in MCCH simulations. In MCCH, if a secondary particle is expected to be produced with an energy below the production threshold, it is not explicitly generated; instead, its energy is deposited locally to improve computational efficiency. In the present database calculations (with MCTS), if a secondary particle was produced with an energy below the MCCH production threshold for that particle type, it was transported, and its ionizations were recorded. This ensures that ionization events from such secondaries are accounted for in the MCCH simulations. They would not be included in MCCH if the same production threshold were applied in the database calculations with MCTS. If a secondary particle had an energy above the production threshold, it was not transported in the database calculation, as its ionizations would be accounted for in MCCH using the precalculated (database) values for its particle class. With this approach there is no double counting of ionization events in MCCH simulations. The production threshold values used in the database calculation were 0.054 MeV for electrons and 1.56 MeV for protons, corresponding to the default production cuts in TOPAS (MCCH simulations) for water. Secondary particles heavier than protons were terminated (MCTS simulations) because the minimum valid energy for MCTS transport is 0.5 MeV u^−1^, and such particles will be transported to lower energies in the MCCH codes used (see physics lists employed in section 2.3). In previously computed databases (Faddegon *et al* 2023), ionizations from all secondary particles were included in the database calculations, regardless of their energy. The ionizations along the entire primary particle track through the simulation volume were considered, without imposing energy loss restrictions.

**Figure 2.**
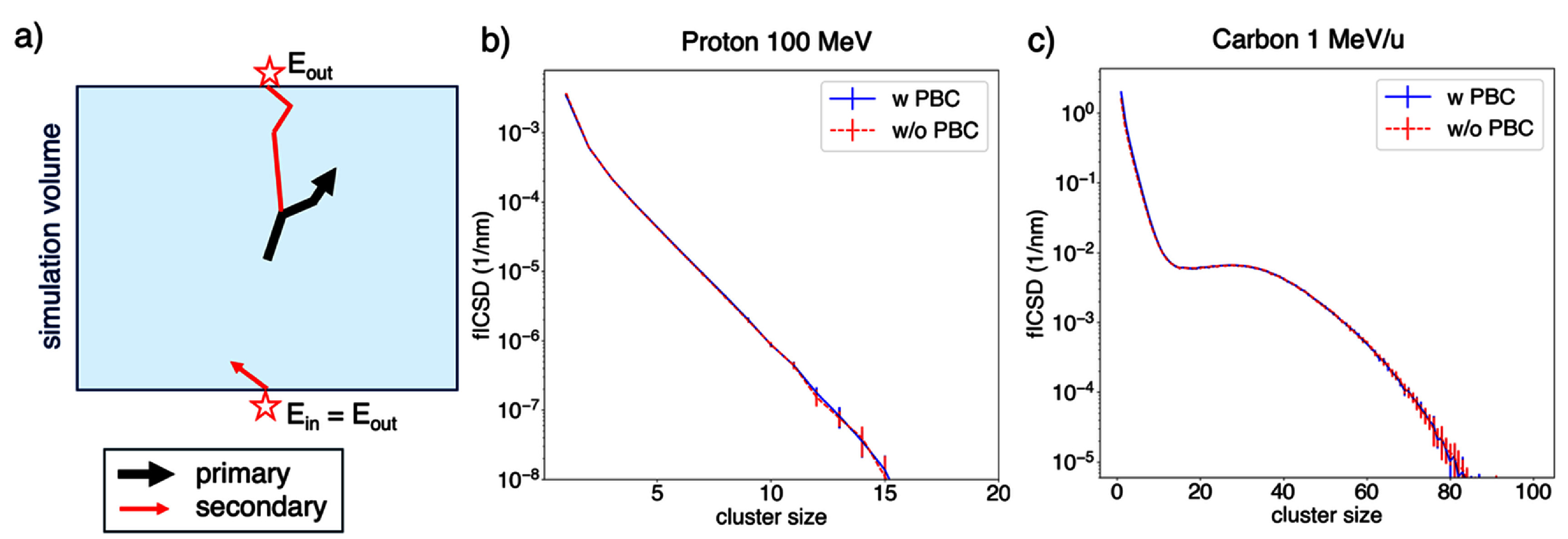
(a) Graphical representation of the implementation of periodic boundary conditions: if a secondary particle (red line segments), generated by a primary particle (black line segments), reaches the boundary of the simulation volume, it is reintroduced to the simulation volume at diametrically opposed point from the exiting point (both represented by red stars) with same energy and direction. fICSD for 100 MeV protons (b) and (a) 1 MeV u^−1^ C ions with and without applying periodic boundary condition to the physical tracks of secondary particles.

To accurately capture all of the ionization due to a primary particle, it is essential to transport each secondary particle along its entire track. This is not the case if portions of tracks escaping the simulation volume are not recorded. In this work, we evaluated the relevance of contributions from charged secondary particles that escape the volume by comparing fICSD values obtained with and without the application of PBCs. Briefly, when a secondary particle reached the boundary of a user-defined rectangular envelope (TsBox), it was reintroduced at the diametrically opposite side with the same energy and direction (see figure 2(a)). This approach enabled tracking of secondaries until they stopped within the simulation volume, without requiring an excessively large geometry that would inordinately increase computation time.

We found that applying PBC to the tracks of secondary particles had no effect on fICSD. This indicates that only a negligible or nil number of particles escape the simulation volume. This may be explained due to the large simulation volume employed, the placement of the radiation source at the center of the simulation volume and the termination of primary particles based on their energy loss, as described in detail above. Figure 2(b) compares fICSD for low-LET protons (100 MeV) and high-LET carbon ions (1 MeV u^−1^), obtained with and without PBC, and shows qualitatively that their use neither increases cluster sizes nor alters cluster frequencies. Therefore, in the database simulations, PBC were not applied, enabling the inclusion of pre-chemistry processes, not compatible with the current PBC implementation in TOPAS.

### 2.3. Macroscale calculation of nanodosimetric quantities

Previous approaches to retrieve nanodosimetric quantities in the patient-level using look-up table in MCCH simulations were as follows. At each simulation step, the particle type and its energy were evaluated in the prestep. This information was then used to retrieve the corresponding ID quantity (fICSD or *I*_p_) from the database by interpolation between database energy values. If the prestep energy was lower or higher than the minimum or maximum energy in the database, the ID quantity associated with the nearest boundary value (i.e. the lowest or highest available energy) was used. That approach did not account for the resolution of database energies nor the change of energy (i.e. ID quantity) along the step.

We propose a revised approach for computing macroscopic ID quantities, as defined in section 2.1, consistent with the database calculation approach.

**Figure 3.**
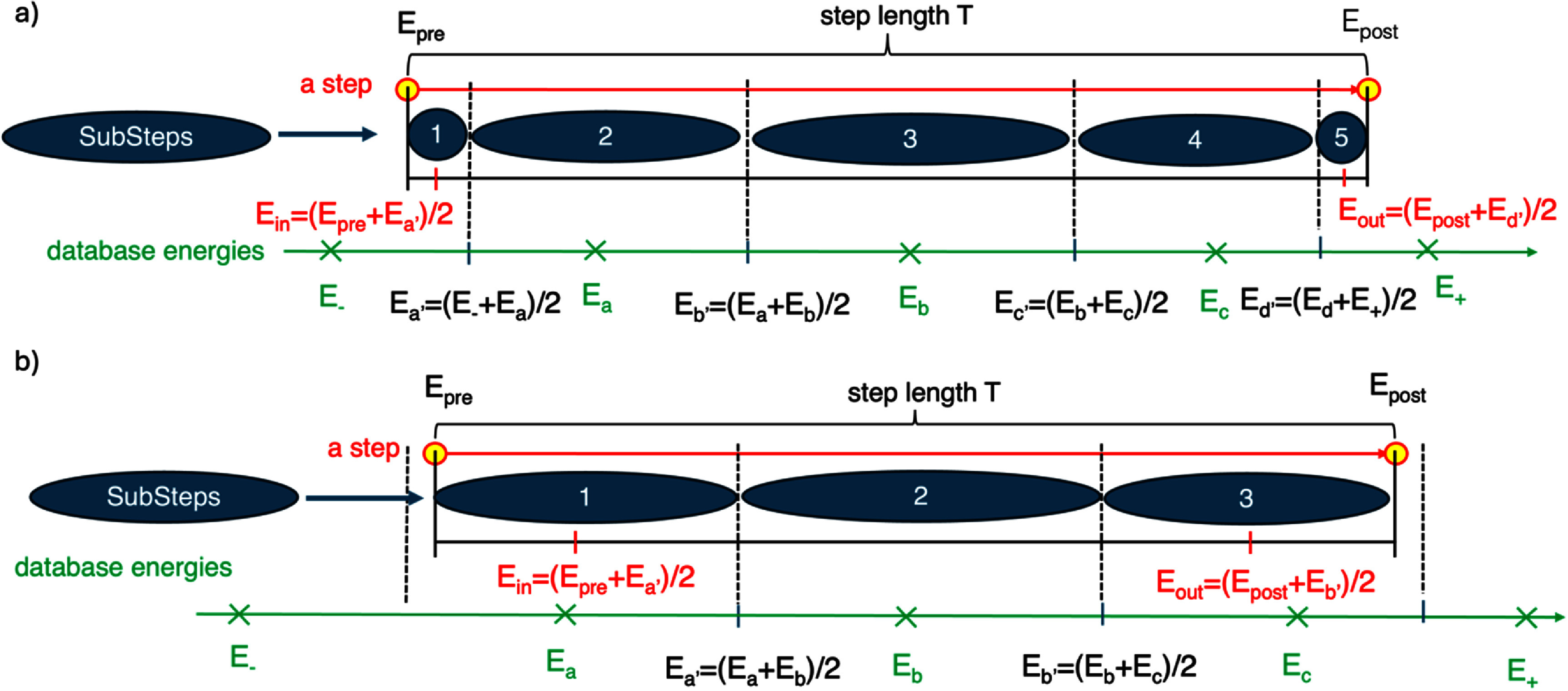
Substeps used in the selection of the precalculated ID database energy, using mid-point energies (*E*_a’_, *E*_b’_, etc.) within the prestep energy *E*_pre_ and poststep energy *E*_post_, showing two cases: (a) three database energies within the step, leading to five substeps, and (b) three database energies within the step, leading to three substeps.

At each step, the prestep energy and poststep energy are evaluated, and the database energies lying between these values identified. The midpoints (*E_x’_*) between adjacent database energies are then used to divide the step into substeps, as illustrated in figure 3.

The energy at the midpoint of each substep is used to calculate the ID quantity (fICSD or *I*_p_). For each substep, both the ID quantity and corresponding track length are calculated as described in table 2, under the assumption of constant energy loss per unit track length (i.e. constant restricted collisional stopping power) throughout the step.

**Table 2.** Computation of ID quantities and track length of each of the *N* substeps.

SubstepID	ID quantity	Track length
1	${I_{\text{p}}} = {I_{\text{p}}}\left( {{E_{{\text{in}}}}} \right)$ is linearly interpolated between database energies, with ${E_{\text{in}}} = \left( {{E_{\text{pre}}} + {\text{ }}{E_{\text{a}^{\prime}}}{\text{ }}} \right)/2{\text{ }}$	$t = \frac{{{E_{\text{pre}}} - {E_{a^{\prime}}}}}{{{E_{\text{pre}}} - {E_{\text{post}}}{\text{ }}}}T$
*n*	${I_{\text{p}}} = {I_{\text{p}}}\left( {{E_n}} \right)$ with $n = a,b,{\text{etc}} \ldots $, is not interpolated since ${E_n}$ is a database energy.	$t = \frac{{{E_{n^{\prime}}} - {E_{\left( {n + 1} \right)^{\prime}}}}}{{{E_{\text{pre}}} - {E_{\text{post}}}{\text{ }}}}T$
*N*	${I_{\text{p}}} = {I_{\text{p}}}\left( {{E_{{\text{out}}}}} \right)$ is linearly interpolated between database energies, with ${E_{{\text{out}}}} = \left( {{E_z}^{\prime} + {E_{{\text{post}}}}} \right)/2$	$t = \frac{{{E_{z^{\prime}}} - {E_{\text{post}}}}}{{{E_{\text{pre}}} - {E_{\text{post}}}{\text{ }}}}T$

This approach accounts for variations in *I*_p_ (or fICSD) across the energy range of a step, which may span multiple energy intervals represented in the database, especially at the end of the particle range, as shown in the Results section. By using substeps centered on energies present in the database, this method minimizes the need for interpolation at intermediate energies and associated interpolation uncertainty. Macroscopic simulations using large step sizes could be subdivided employing stopping power for the track length calculation should the assumption of constant stopping power along the step introduce significant error.

This approach was implemented in a developer code of TOPAS, version OpenTOPAS v.4.0 (Geant4-11.1.3). For calculations of voxel-averaged *I*_p_ and cluster dose, standard Geant4 physics cross-section data files from the physics list built using the Geant4_Modular option with ‘g4em-standard_opt4’, ‘g4h-phy_QGSP_BIC_HP’, ‘g4decay’, ‘g4ion-binarycascade’, ‘g4h-elastic_HP’, ‘g4stopping’ modules were used. All transport parameters were set to default values, and the production threshold for secondary particles was defined by the energy cutoff used in the database calculations (see section 2.2.3).

## 3. Results

### 3.1. Database quantities

**Figure 4.**
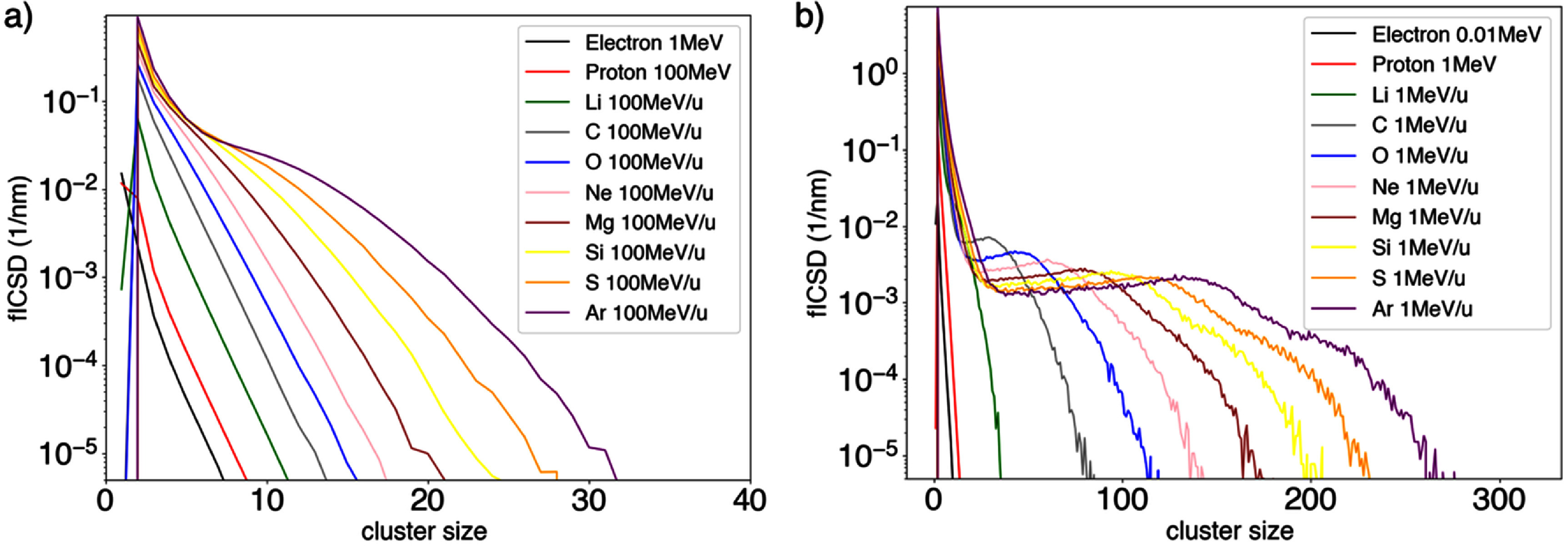
fICSD in the database presented in this work for (a) high and (b) low energies for selected particle types. Cluster size (horizontal axis) corresponds to the number of ionization per cluster, and fICSD (vertical axis) is the frequency distribution of ionization cluster sizes (see section 2.1 for more details).

A representative set of fICSD from the database computed in this work for selected particles at different energies is presented in figure 4. Heavier particles have a higher LET, resulting in a higher frequency of larger clusters for the higher energy particles shown in the figure. Additionally, for the lower energy particles, having higher LET, the maximum energy transferred to secondary electrons decreases (Francis *et al* 2011), produce more densely ionizing lower energy electrons, creating a higher number of local ionizations and resulting in more large ionization clusters.

At lower energies and for heavier ions, the frequency of clusters as function of the cluster size exhibited an initial drop, followed by a region of constant values and a slightly increase. This pattern has been previously observed in ionization cluster size probability distributions obtained in computational studies (Ramos-Méndez *et al* 2018, D-Kondo *et al* 2024) and experimental measurements (Hilgers *et al* 2017). This characteristic shape is attributed to the differential contribution from primary ionizations producing large clusters and secondary particle ionizations produced small clusters, as described elsewhere (Ramos-Méndez *et al* 2018). This effect is most pronounced at higher LET (lower energy).

### 3.2. Energy spacing

**Figure 5.**
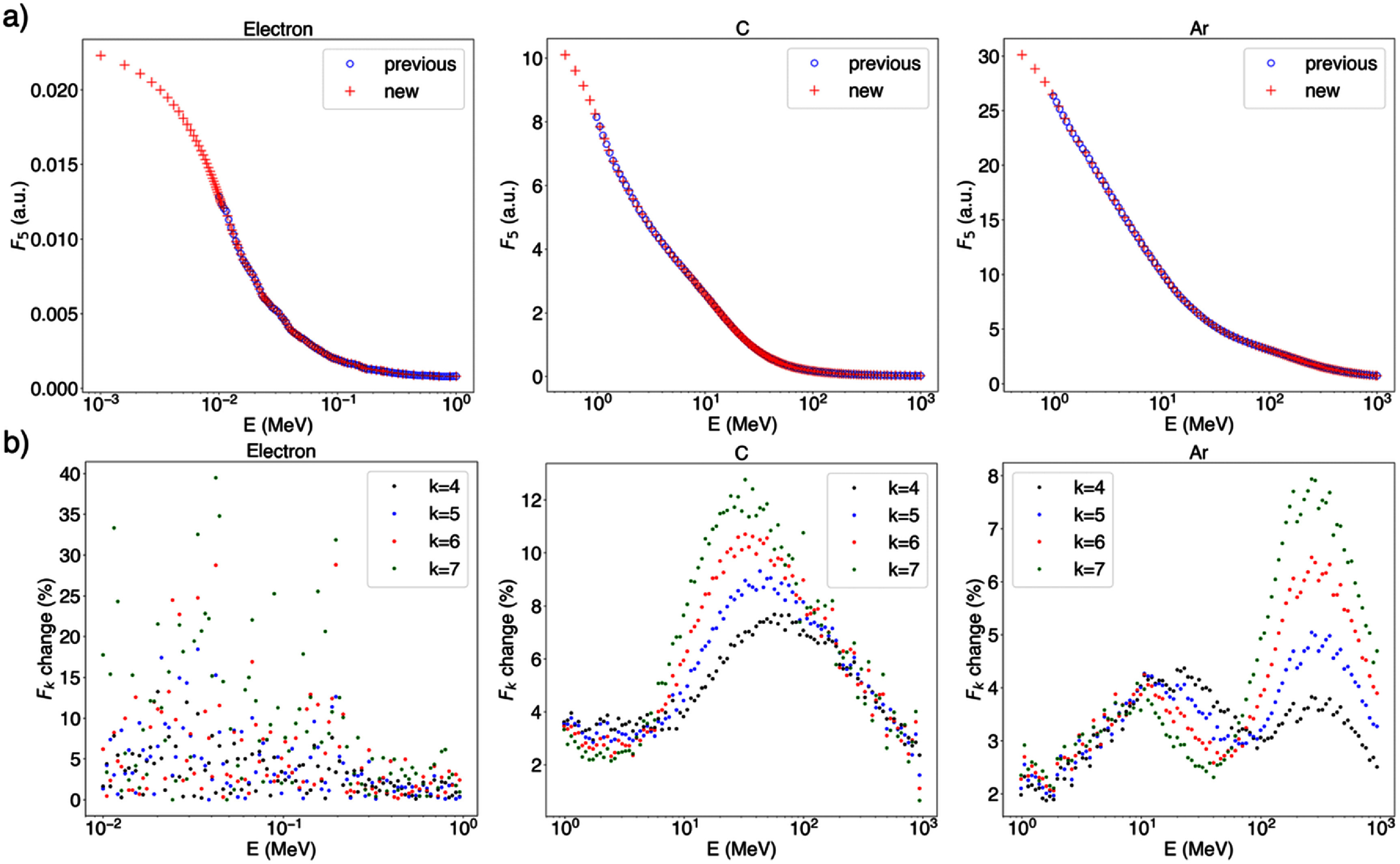
(a) Energy binning in previously computed (blue o) and the present (red +) databases. (b) Variation in *I*_p_ between adjacent energy values in the previous database. The maximum variation with the proposed energy binning is 5%.

The spacing between database energies was chosen for each particle type to maintain a maximum variation of 5% in *F_5_* to *F_7_* between adjacent energies. In contrast to previous database calculations (Faddegon *et al* 2023), where the set of energies consisted of 100 energy bins, evenly spaced on the logarithmic scale, the energy spacing in the present approach is tailored to the specific change of *I*_p_ as function of the energy for each particle type. With previous approaches, variations in *F_5_* to *F_7_* between adjacent energies were as high as 40%. Figure 5 shows the proposed energy binning as compared with previous approaches and the respective change in *I*_p_ for a selected set of particle types.

The proposed energy binning ensures that interpolation errors for energies not explicitly included in the database remain below 1%. For demonstration, ID quantities were computed for three randomly sampled energies per particle within the ranges listed in table 1. The difference between explicitly computed and interpolated F_5_ to F_7_ values was assessed, and in all tested cases, the interpolation error remained within the 1% standard deviation of the computed values.

### 3.3. Isotopes

**Figure 6.**
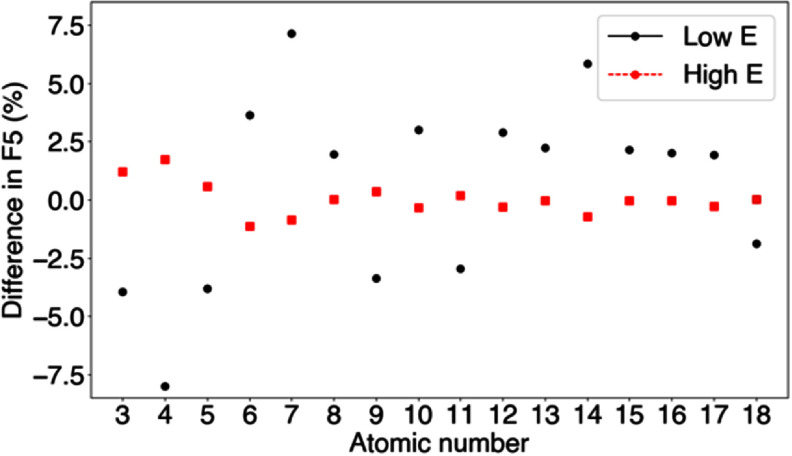
Mean difference in F_5_ for different isotopes as function of the atomic number for the lowest and highest energies presented in table 1.

The proposed database was limited to ions from stable elements. To consider the differences in nanodosimetric quantities between different isotopes, *I*_p_ values were calculated for a set of potentially clinically relevant isotopes: 6-Li, 7-Be, 10-B, 13C, 15-N, 15-O, 18-F, 21-Ne, 22-Na, 25-Mg, 28-Al, 29-Si, 32-P, 33-S, 36-Cl, and 39-Ar. We compared their nanodosimetric quantities against those from stable ions for the lowest and highest energies for each particle type (each atomic number) presented in table 1. The difference in *F_k_*, calculated as the relative difference between the isotope and the stable ion—$100*(F_{\text{k}}^{\text{isotope}} - {\text{ }}F_{\text{k}}^{\text{stable}})/F_{\text{k}}^{\text{stable}}$ —is shown in figure 6. The variation in *F_5_* and *F_7_* between isotopes was within 8%, with an average absolute variation of 2% across all tested atomic numbers.

To assess the clinical impact of these differences, the isotope yield, defined as the percentage of unstable isotopes relative to the total number of charged particles at the distal edge of proton, carbon, and oxygen ion SOBPs, was quantified. The isotope yield varied between ∼0% and 14%. Consequently, in macroscopic calculations, the use a single fICSD per atomic number and applying it uniformly to different isotopes, the expected variation in relevant *I*_p_ definitions (i.e. *F_5_* and F_7_) is expected to be within 1%, ⩽ 0.3% on average.

### 3.4. Comparison with previous ID databases

**Figure 7.**
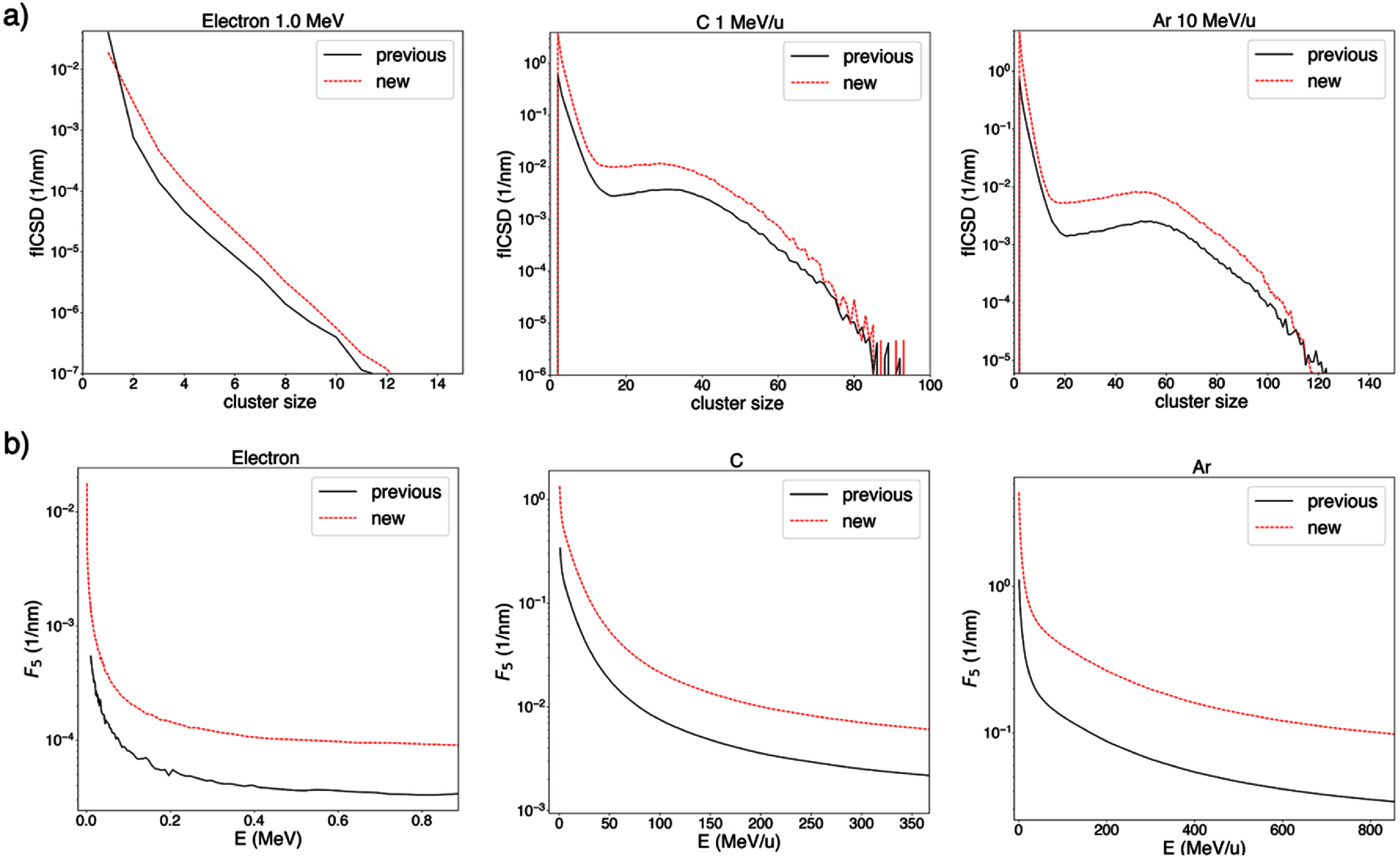
(a) fICSD for selected particle classes and (b) *I*_p_ as function of particle energy in previously computed and currently proposed database.

Figure 7 illustrates a comparison of fICSD and *I*_p_ in the previously computed and currently proposed databases for a representative selection of particle classes.

Overall, fICSD values (i.e. the absolute frequency of clusters) are higher with the proposed approach. This increase is primarily due to the greater number of sampling volumes in the simulation volume, as described in section 2.2.1.

**Figure 8.**
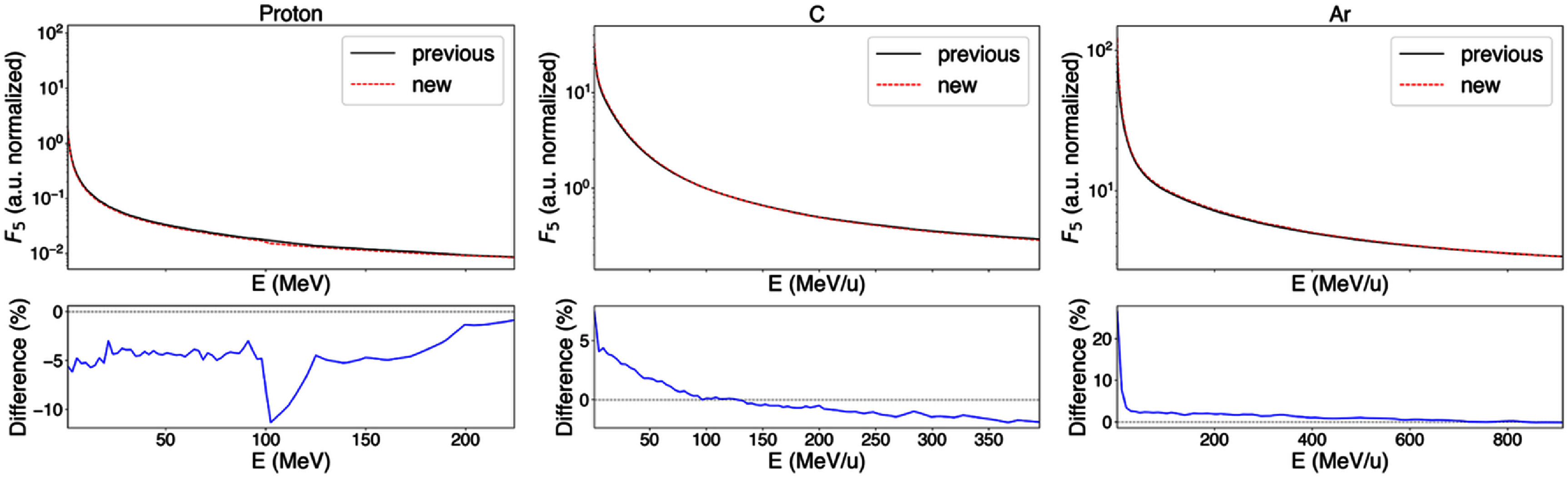
*F_5_* as function of the particle energy for selected particle types normalized to a reference value (*F_5_* of a 100 MeV u^−1^ carbon ion) in previously computed and currently proposed (new) databases. Each reference value is extracted from each respective database.

Treatment plans will be primarily impacted by the change in the relative variation of *I*_p_ and cluster dose between different approaches used to calculate these quantities. When *F_5_* values are compared in terms of their relative change with respect to a reference particle class (100 MeV u^−1^ carbon ion) in each of the databases, differences in the relative variation of *F_5_* between the two databases of up to 10% for protons, 7% for C ions and 25% for Ar ions are observed (see figure 8). This is attributed to the different treatment of secondary particles in the two approaches.

### 3.5. Macroscale scoring

**Figure 9.**
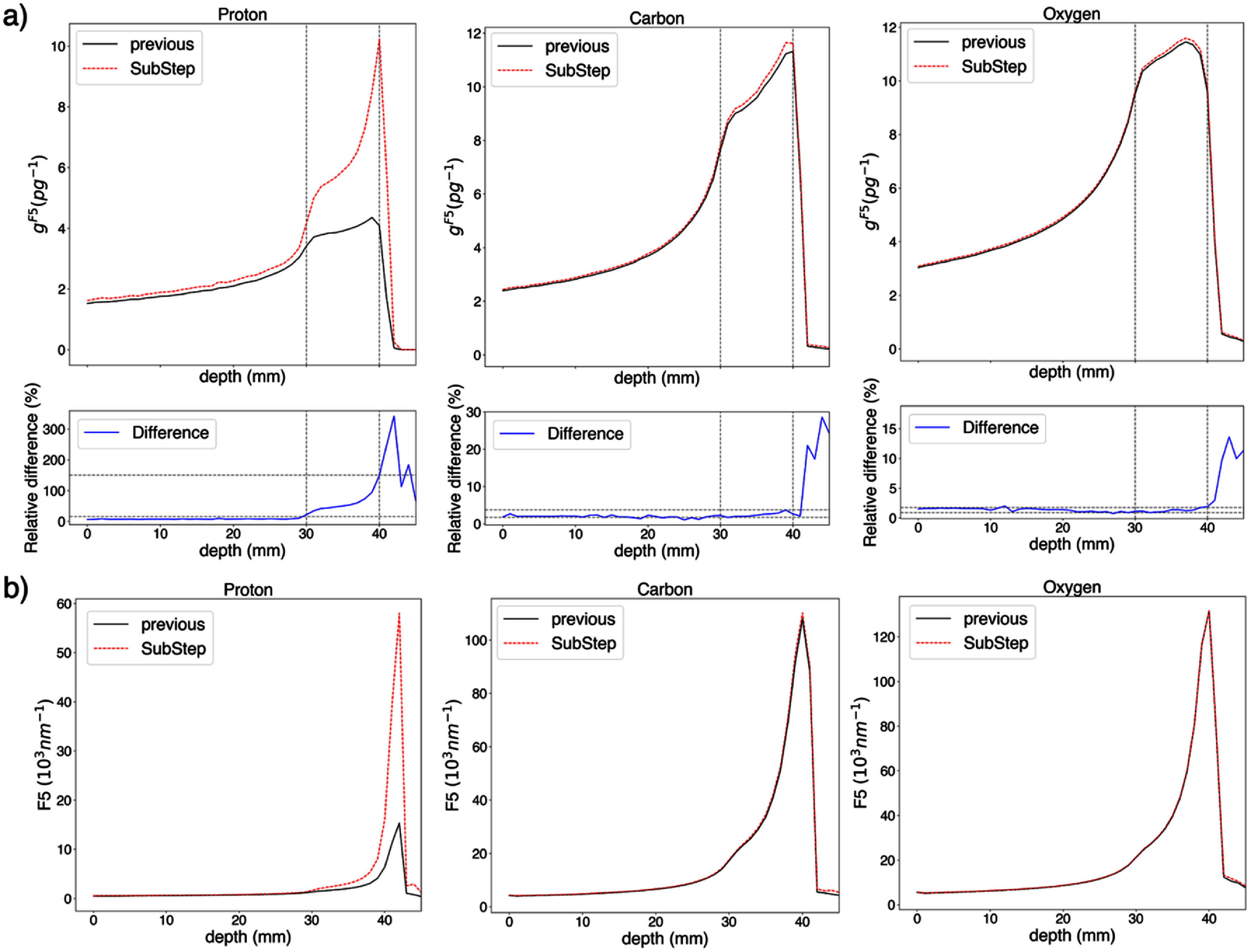
(a) Cluster dose and (b) *F*_k_ dose curves of proton, C and O ion SOBP computed using the previously and currently proposed (substep) approaches for macroscale computation of ID quantities, using the new database. Dashed grey vertical lines indicate the SOBP width (i.e. from 3 to 4 cm) and dashed grey horizontal lines the relative difference at these depths.

Figure 9 presents the differences in voxel-averaged *I*_p_ and cluster dose depth profiles when applying the substep approach to scoring in MCCH simulations, compared to previous methods that assigned a single *I*_p_ (or fICSD) value to the entire step based on the prestep energy. Proton, carbon, and oxygen ion SOBP configurations from the experimental beamline at the Heidelberg Ion Therapy (HIT) Center (Dokic *et al* 2016) were used for demonstration purposes. Experimental setups and beam information were reproduced to match published experimental absorbed dose distributions, created using a methodology reported elsewhere (Ortiz and Faddegon 2024).

**Figure 10.**
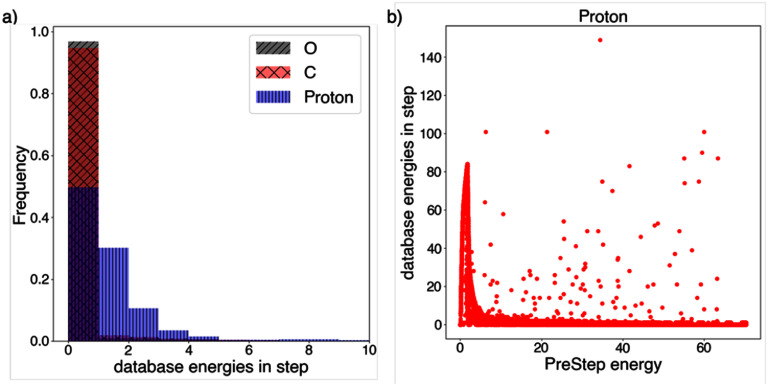
(a) Histogram showing the number of database energy intervals spanned within MCCH steps for proton, carbon (C), and oxygen (O) in the SOBP configurations reported in figure 9. (b) Number of database energies per step as a function of the prestep energy for the proton SOBP case.

Overall, an increase in *F_5_* is observed at the distal edge of the SOBP, with over two-fold increase in cluster dose within the SOBP and three-fold increase distal to the SOBP for protons, up to 3% increase within the SOBP and 30% at the distal edge for carbon ions, and up to 2% and 10% within the SOBP and at the distal edge, respectively, for oxygen ions. The new scoring approach at the macroscale, i.e. subdividing each step into multiple substeps, has a greater influence on the proton cluster dose distributions as compared to heavier ions since the energy loss within a single Monte Carlo step spans energy intervals represented in the database (see figure 10(a)). This effect is larger at the distal edge of the SOBP since the number of energy intervals per step is greater at lower proton energies near the end of the particle range (see figure 10(b)).

### 3.6. Impact on cluster dose distributions

**Figure 11.**
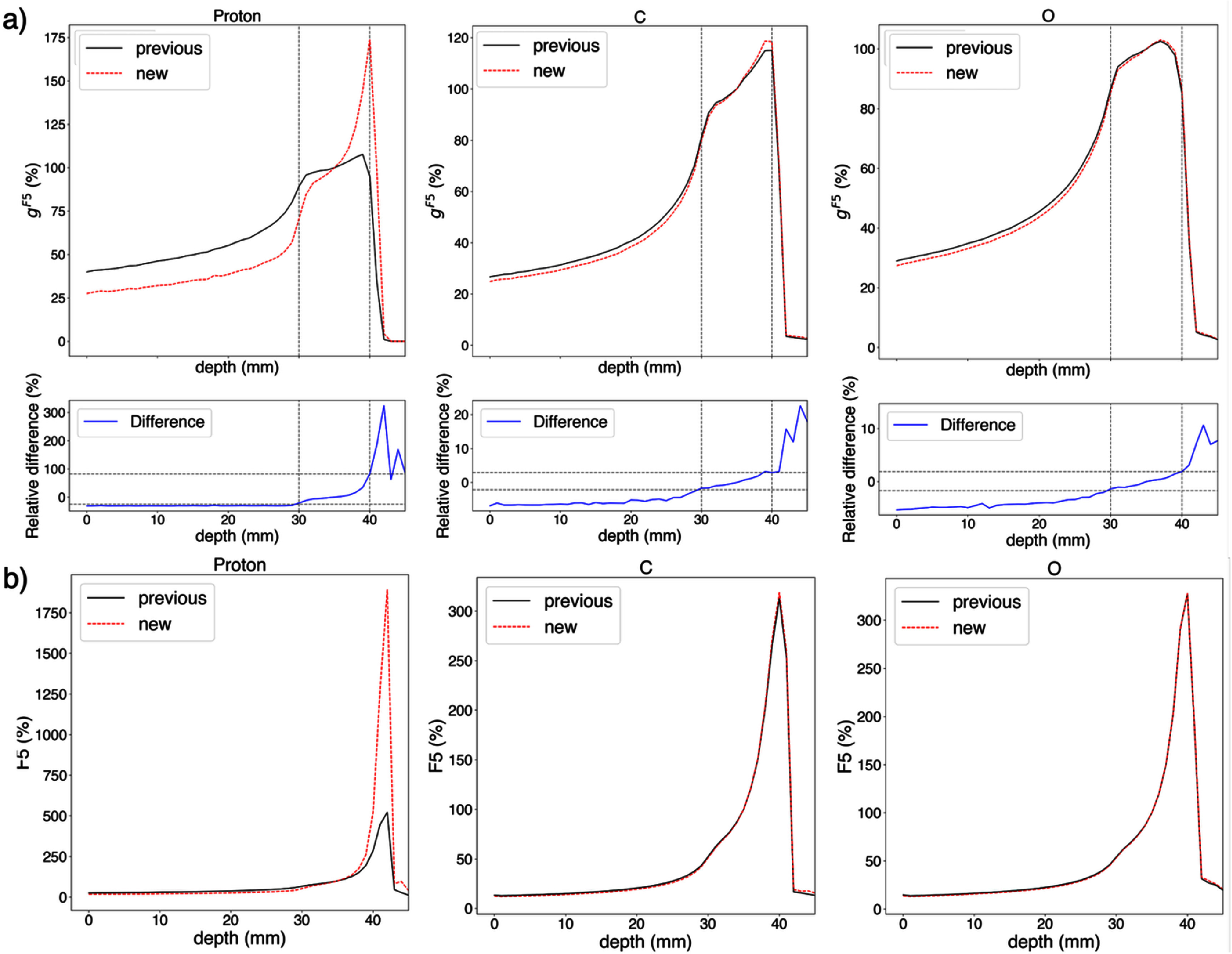
(a) Cluster dose and (b) *F*_k_ dose curves of proton, C and O ion SOBP computed using the previously and currently proposed approaches at nanoscale and macroscale. Dashed grey vertical lines indicate the SOBP width (i.e. from 3 to 4 cm) and dashed grey horizontal lines the relative difference at these depths.

The combined impact of both the proposed database computation and macroscale scoring approaches on voxel-averaged *I*_p_ and cluster dose distributions at the macroscopic scale (i.e. MCCH calculations) was investigated for proton, carbon, and oxygen ion SOBP configurations as follows. Relative depth cluster dose curves, normalized at the center of the SOBP (3.5 cm in water), were compared using both approaches. As shown in figure 11, relative differences in cluster dose of up 100% were observed for protons within the SOBP and three times at the distal edge, up to 7% for carbon ions at the plateau region and 3% within the SOBP and 20% at the distal edge, and up to 5% for oxygen ions at the plateau, 2% within the SOBP and 10% at the distal edge. These differences are attributed to variations in the relative *F*_k_ values across particle types and energies contributing to the voxel-averaged *I*_p_ along the depth curves, as described in section 3.4. and the impact of accounting for *I*_p_ variation along each step in the proposed macroscale scoring approach, as discussed in section 3.5.

### 3.7. Impact on the association of nanodosimetric quantities with cell survival

**Figure 12.**
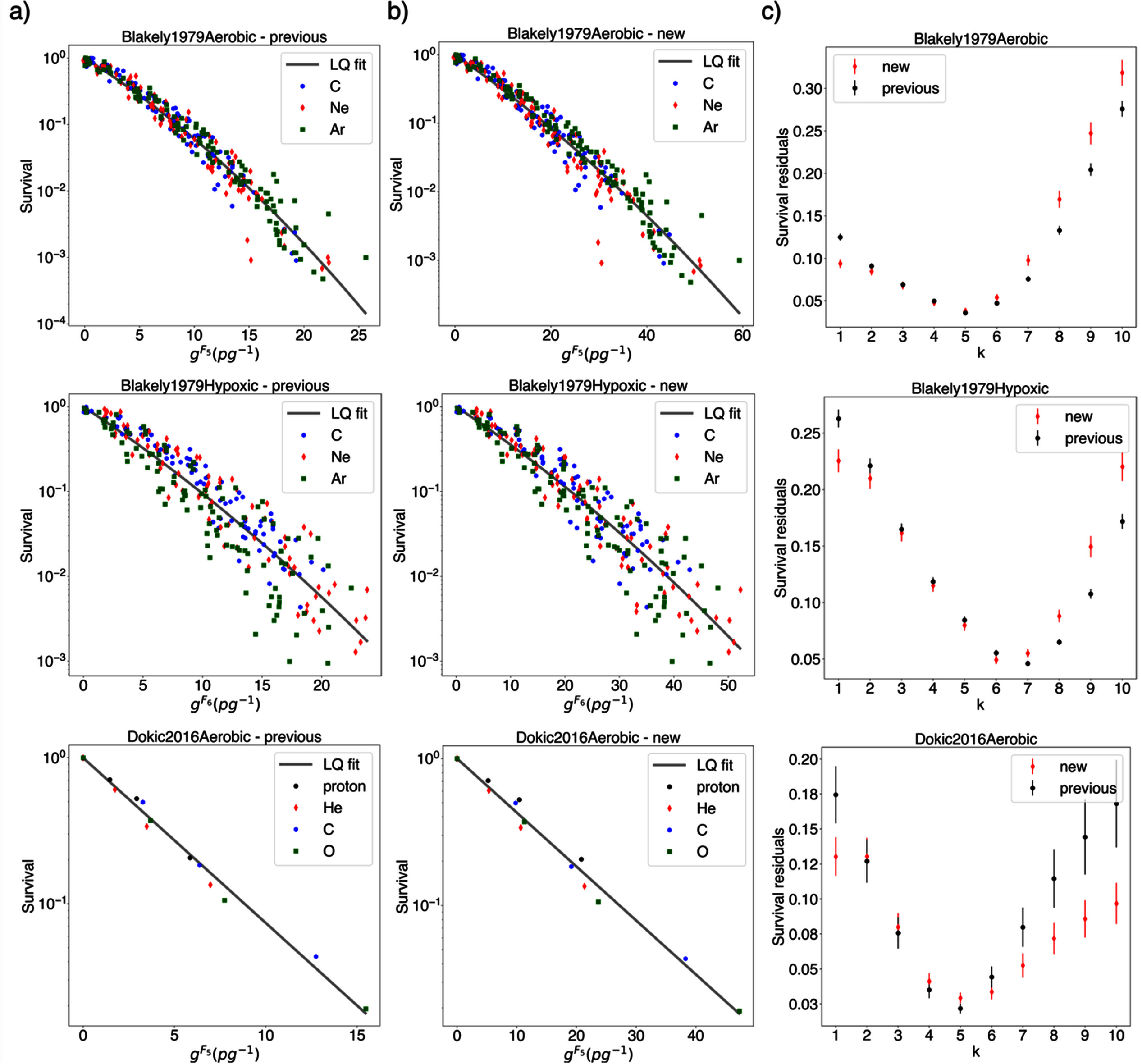
Cluster dose survival curves in three different datasets calculated using the (a) previously and (b) currently proposed databases. (c) Survival residuals from a common LQ fit to each dataset in both cases.

Differences in *I*_p_ and cluster dose depth curves can influence the selection of the preferred *I*_p_. One method for determining the preferred *I*_p_ is based on the correlation between cluster dose and cell survival, as analyzed through cluster dose survival curves (i.e. survival plotted against cluster dose), as detailed in our previous work (Ortiz *et al* 2025). As described in section 1, the preferred *I*_p_ is defined as the one for which cell survival remains nearly identical at the same cluster dose independently of particle types and energies. Variations in cluster dose depth distributions due to differences in database computations will alter the cluster dose received by a cell sample at a given position, thereby affecting the cluster dose survival curves. To illustrate how the selection of the preferred *I*_p_ may vary when using the refined approaches presented in this work with respect to previous approaches, we examined three datasets from previous studies on preliminary *I*_p_ selection (Faddegon *et al* 2023, Ortiz *et al* 2025). Two datasets include survival measurements of human kidney T-1 cells obtained at Lawrence Berkeley National Laboratory (LBNL) at various positions along the Bragg curves of carbon, neon, and argon beams, with initial energies of 400, 425, and 570 MeV u^−1^, respectively, under aerobic and hypoxic conditions (Blakely *et al* 1979), referred as *Blakely1979Aerobic* and Blakely1979Hypoxic hereafter. The third dataset contain survival data of human alveolar adenocarcinoma A549 cells at the center of a 1 cm proton, helium, carbon and oxygen SOBP, measured at the HIT center (Dokic *et al* 2016), referred as *Dokic2016Aerobic* hereafter. Figure 12 presents the variations in cluster dose survival curves and the selection of the preferred *I*_p_ using survival residuals from a LQ fit to each dataset, comparing results from the previously computed and the currently proposed approaches. The *I*_p_ that leads to the lowest mean residual across the dataset is considered the preferred *I*_p_ definition (Ortiz *et al* 2025). Differences in cluster dose survival curves are observed between the use of the two databases. Although the selection of the preferred *I*_p_, defined as the *I*_p_ yielding the lowest survival residuals from a common fit, remains unchanged for two of the datasets (Blakely1979Aerobic and Dokic2016Aerobic), corresponding to *F_5_*, respectively, the relative difference between *I*_p_ definitions is affected, as shown in figure 12(c). In contrast, for the third dataset (Blakely1979Hypoxic) the new approach leads to the identification of *F_6_* as the preferred Ip, in contrast to the selection of *F_7_* with the previous methodologies.

## 4. Discussion

Nanodosimetric quantities have already demonstrated their potential to improve ion RTP. The ID formalism proposes the use of cluster dose, a macroscopic physical quantity derived from nanodosimetric ID parameters, that scales with fluence, enabling its integration into RTP workflows (Faddegon *et al* 2023). Previous studies have shown that, for preferred definitions of *I*_p_, such as *F_5_* and *F_7_* under aerobic and hypoxic conditions, an equivalent cluster dose yields comparable cell survival independent of particle type, energy, and fluence (Ortiz *et al* 2025).

To apply nanodosimetric quantities computed at the nanoscale using MCTS simulations to the patient level via macroscopic quantities such as cluster dose (computed using MCCH simulations), several considerations must be addressed to reconcile both radiation transport simulation methods and provide seamless transition between the different scales. This work proposes a robust methodology for generating a comprehensive database of nanodosimetric quantities and for integrating them into macroscale scoring methods. Compared to previous approaches, the proposed method improves accuracy by extending the energy range of the database, refining energy binning, and ensuring consistent treatment of secondary particles. Additionally, a revised macroscale scoring method was introduced to account for energy variations along particle tracks, consistent with database calculations. In this study, we employed validated Monte Carlo codes for radiation transport at nanometric scales (Incerti *et al* 2010, Bernal *et al* 2015, Burigo *et al* 2016, McNamara *et al* 2017, Masilela *et al* 2025). Published experiments for benchmarking at this scale differ substantially from the present study, e.g. (Pszona *et al* 2000, Garty *et al* 2002, Conte *et al* 2012, Bantsar *et al* 2015, Hilgers *et al* 2017, Selva *et al* 2020). The geometries and transport considerations in this work were specifically designed to enable seamless integration of nanodosimetric quantities into macroscale calculations for RTP applications. A direct comparison with experimental data would require either alternative simulation setups tailored to reproduce specific experimental conditions or new experiments explicitly designed to match the considerations adopted here, which lies beyond the scope of the present work.

The adaptive energy binning introduced in this work offers a refinement over previous methods by considering the change of *I*_p_ as function of energy for each particle type. This results in a more efficient and accurate database structure, reducing the resolution where *I*_p_ varies slowly and increasing resolution where needed to obtain a maximum change of 5% in *I*_p_ between adjacent energies. In macroscale calculations for treatment planning, the particle energy at interaction points is governed by the algorithms and physics models implemented in MCCH simulations and treatment planning systems (Facchiano *et al* 2025). The refined binning guarantees that, regardless of the energy dictated by these models, interpolation from the database yields nanodosimetric quantities with errors below 1%. This refinement is particularly relevant for voxel-averaged *I*_p_ and cluster dose calculation in MCCH simulations, where the energy at a given step (or substep) may not exactly match a value listed in the database and where linear interpolation is used for the estimation of the *I*_p_. In addition, as described in section 2.3, the new macroscale scoring approach subdivides each step into multiple substeps according to the available database energies. By employing refined binning, the substep division is optimally aligned with the maximum variation in *I*_p_ allowed between adjacent database energies, ensuring more precise voxel-level scoring.

The ID database was only calculated for stable ions present in the periodic table. For different isotopes of a given ion, the corresponding ID quantity from the stable isotope is applied. In this work, we studied the impact of this approximation. Differences in ID quantities between the different isotopes studied can reach up to 8%, with an average absolute variation of ∼2% (figure 6). The observed differences between isotopes are attributed to the mass/energy scaling of the cross-section for heavy ions above alpha particles, without consideration of charge states, which are not included in the Geant4 cross-sections for these particles. Given the relatively low abundance of isotopes in clinical scenarios (between 0%–14%), the expected impact of the use of a single ID quantity for the different isotopes of a given ion on cluster dose calculations remain low (⩽0.3%). Therefore, the use of a single ID quantity per atomic number is expected to have negligible impact in clinical cluster dose distributions.

Comparison between the databases computed in this work and in previous studies shows an overall increase in fICSD and *F*_k_ values with the present approach (figure 7). This is primarily attributed to the use of a greater number of sampling volumes, which improves statistical accuracy and enhances sensitivity to ionization events, as described in section 2.2.1.

Applying PBCs within the simulation volume had a negligible impact on fICSD, as shown in figure 2 for both low-LET (100 MeV protons) and high-LET (1 MeV carbon ions); that is, a negligible fraction of clusters were deposited by the physical track of secondary particle outside the simulation volume. This results from using a sufficiently large simulation volume, a small, spherical isotropic radiation source placed at the center of the simulation volume, and the application of a limit of energy loss for primary particles to be terminated (see section 2.3). Since the current TOPAS implementation of PBC is only compatible with physical processes, omitting them allowed us to score ionization at the point of creation of ionized species (pre-chemical stage), without producing potential artifacts in ID quantities arising from missed contributions of escaping particles.

Differences of up to 20% in the relative change in *I*_p_ with respect to a reference particle class (100 MeV u^−1^ carbon ions) were observed (figure 9). This highlights the differences in the inclusion of the contribution of secondary particles to ID quantities between the proposed and previous approaches. In previous approaches the inclusion of secondary particles varied across particle classes, i.e. no energy cutoff was applied. In contrast, the proposed approach has been verified to incorporate the full contribution of secondary particles along their range, as confirmed by the analysis of PBC described above, and applies a common energy cutoff, ensuring consistent secondary particle treatment across all particle classes.

Regarding the revised macroscale scoring (i.e. the substep approach), this approach leads to an increase in voxel-averaged *I*_p_ and cluster dose as compared to previous approaches at the end of the particle range. This increase arises because, in previous approaches, the entire step was characterized by a single *I*_p_ value corresponding to the prestep energy, even though the particle’s energy could span multiple database intervals within that step, as shown in figure 10. In contrast, the substep method accounts for energy loss along the step and corresponding change in *I*_p_. Since lower energies are associated with higher *I*_p_ values, this results in higher *I*_p_ values near the end of the step. This effect is more pronounced at lower energies, i.e. toward the distal end of the particle range. In addition, because substeps are defined by the particle energies included in the database, no interpolation is required for intermediate energies, minimizing potential interpolation errors.

Overall, the refined database calculation and revised macroscale scoring approach affect significantly cluster dose distributions, with relative differences in depth distributions of up 300% for protons, 20% for carbon ions, and 10% for oxygen ions in tested SOBPs (figure 11). Overall, the approach presented in this work ensures that the contribution of all primary and secondary particles is consistently accounted for in the calculation of cluster dose while avoiding double counting of ionizations by applying a common energy cutoff for the inclusion of secondary particles in the database. This energy cutoff can be directly used in MCCH simulations to define the production threshold for secondary particles. Specifically, if the ionizations produced by a secondary particle are included in the database (i.e. the production energy is below the cutoff), its ionizations are not explicitly added to the voxel-averaged *I*_p_ in MCCH simulations, as they are already accounted for through the primary particle’s contribution. If the secondary particle is not included in the database (i.e. its energy is above the cutoff), it is explicitly tracked in MCCH simulations, and its contribution to voxel-averaged *I*_p_ and cluster dose is accounted for using the corresponding database values for that particle class. This approach ensures a consistent and systematic treatment of secondary contributions to *I*_p_ and cluster dose in MCCH simulations at patient level. In contrast, the previous approach lacked a common energy cutoff, which may lead to inconsistencies in how secondaries were accounted for and inaccuracies in macroscopic calculations for some Ip definitions.

A definition of *I*_p_ suitable for ID-based RTP would result in an increase in cluster dose comparable to the increase in relative biological effectiveness, with predictions ranging from unity to a factor of three (Gardner *et al* 2024). The large increase in the F_5_ cluster dose across the proton cluster dose SOBP in figure 11 may be observed in radiobiological datasets used to identify preferred *I*_p_. A much smaller increase in cluster dose is achieved by including clusters of two to four ionizations in the cluster dose, with these smaller clusters weighted with a reduced probability of cell kill compared to larger clusters. Thus, the magnitude of the increase in the F_5_ cluster dose across the proton cluster dose SOBP seen with the new database and calculation approach is not a cause for concern in using cluster dose for ID-based RTP.

In the macroscale scoring process, i.e. voxel-averaged *I*_p_ computation in MCCH, nanodosimetric quantities for particles with energies outside the database range are assigned the values corresponding to the lowest or highest database energies. In the approach proposed in this work, the energy range was extended at lower energies i.e. from 10 keV to 1 keV for electrons, from 0.5 MeV to 100 keV for protons, and from 1 MeV u^−1^ to 0.5 MeV u^−1^ for heavier ions. The explicit computation of nanodosimetric quantities at these lower energies and their application to voxel-averaged *I*_p_ calculations can also contribute to the differences observed. Additionally, the previous approach exhibited larger differences in *I*_p_ values between adjacent database energies, as discussed in section 3.2. These larger gaps could introduce interpolation inaccuracies for energies not explicitly included in the database, therefore affecting cluster dose distributions. The refined energy binning in the current approach, along with the proposed macroscale scoring approach, mitigates these effects, leading to a more accurate representation of ID quantities across all relevant energies.

Differences in *I*_p_ and cluster dose distributions can impact the selection of the preferred *I*_p_, which is determined based on the ability of the cluster dose to predict equivalent levels of cell survival across different particle types and energies. In this work, we studied the impact of the new approaches on the association of cluster dose with cell survival. For that, we selected three datasets covering a wide range of LET and *I*_p_ values, for both research and clinical beam, with two different cell lines under aerobic and hypoxic conditions. The experiments were conducted by different researchers at two independent institutions. Our results show that variations in cluster dose distributions between the previous and current databases can alter the relevance of different *I*_p_ definitions (figure 12). The new approaches introduced in this work affected the selection of the preferred *I*_p_ for one of the dataset studied (Blakely1979Hypoxic), resulting in the selection of a different preferred *I*_p_, where the revised methodology identified *F_6_* as the preferred *I*_p_, in contrast to *F_7_* obtained with previous methods. These findings highlight the importance of the more accurate methodologies developed in this study for transitioning from the nanoscale to the macroscale, as they directly impact both the selection of the preferred *I*_p_ and the derived definition of cluster dose. A detailed evaluation of the biological rationale behind the selection of preferred *I*_p_ definitions is beyond the scope of the present work and warrants a separate study with a larger set of biological data. This is the focus of ongoing work designed to identify preferred *I*_p_ definitions. These studies involve large sets of published cell survival experiments covering diverse cell lines, with sufficient charged particle types and/or energies to contribute to the assessment, providing the beam and experimental set-up details required for accurate calculation of the ID parameters. The establishment of preferred *I*_p_ for ID-based RTP will further benefit from a wide set of quality data with a variety of biological endpoints closely aligned with radiotherapy outcome. These investigations are expected to benefit from the ID database and macroscale scoring approach established in this work.

The database and cluster dose calculation method are also intended to support future studies on ion RTP using cluster dose. While the macroscale scorer used in this study has been implemented in TOPAS, the proposed methodology can also be adapted for its use in other calculation frameworks better suited for clinical RTP applications, such as GPU-based Monte Carlo codes or fast pencil-beam algorithms.

The consistent methodology developed here for computing nanodosimetric quantities may also facilitate more meaningful comparisons with microdosimetric quantities of relevance in radiation therapy, given their mutual association with biological endpoints.

## 5. Conclusion

This work presents a database of nanodosimetric ID quantities, tailored for use in ID-based RTP applications. This database, together with the updated scoring methodology, facilitates the integration of nanodosimetric quantities into macroscopic calculations, providing the means for biologically optimized ion therapy based on cluster dose.

## Data Availability

The ID database will be available through an agreement with the University of California, San Francisco (UCSF). Please reach out to Innovation@ucsf.edu referencing SF2025-035 with a request for access.

## References

[pmbae2230bib1] Bantsar A, Hilgers G, Pszona S, Rabus H, Szeflinski Z (2015). Experimental investigation of ionisation track structure of carbon ions at HIL Warsaw. Radiat. Prot. Dosim..

[pmbae2230bib2] Bernal M A (2015). Track structure modeling in liquid water: a review of the Geant4-DNA very low energy extension of the Geant4 Monte Carlo simulation toolkit. Phys. Med..

[pmbae2230bib3] Blakely E A (1992). Cell inactivation by heavy charged particles. Radiat. Environ. Biophys..

[pmbae2230bib4] Blakely E A, Tobias C A, Yang T C, Smith K C, Lyman J T (1979). Inactivation of human kidney cells by high-energy monoenergetic heavy-ion beams. Radiat. Res..

[pmbae2230bib5] Burigo L N, Ramos-Méndez J, Bangert M, Schulte R W, Faddegon B (2019). Simultaneous optimization of RBE-weighted dose and nanometric ionization distributions in treatment planning with carbon ions. Phys. Med. Biol..

[pmbae2230bib6] Burigo L, Pshenichnov I, Mishustin I, Hilgers G, Bleicher M (2016). Distributions of deposited energy and ionization clusters around ion tracks studied with Geant4 toolkit. Phys. Med. Biol..

[pmbae2230bib7] Charlton D E, Nikjoo H, Humm J L (1989). Calculation of initial yields of single- and double-strand breaks in cell nuclei from electrons, protons and alpha particles. Int. J. Radiat. Biol..

[pmbae2230bib8] Chatzipapas K P, Papadimitroulas P, Emfietzoglou D, Kalospyros S A, Hada M, Georgakilas A G, Kagadis G C (2020). Ionizing radiation and complex DNA damage: quantifying the radiobiological damage using Monte Carlo simulations. Cancers.

[pmbae2230bib9] Conte V, Bianchi A, Selva A (2023). Track structure of light ions: the link to radiobiology. Int. J. Mol. Sci..

[pmbae2230bib10] Conte V, Colautti P, Grosswendt B, Moro D, Nardo L D (2012). Track structure of light ions: experiments and simulations. New J. Phys..

[pmbae2230bib11] Conte V, Selva A, Colautti P, Hilgers G, Rabus H (2017). Track structure characterization and its link to radiobiology. Radiat. Meas..

[pmbae2230bib12] Conte V, Selva A, Colautti P, Hilgers G, Rabus H, Bantsar A, Pietrzak M, Pszona S (2018). Nanodosimetry: towards a new concept of radiation quality. Radiat. Prot. Dosim..

[pmbae2230bib13] D-Kondo N, Ortiz R, Faddegon B, Incerti S, Tran H N, Francis Z, Barbosa E M, Schuemann J, Ramos-Méndez J (2024). Lithium inelastic cross-sections and their impact on micro and nano dosimetry of boron neutron capture. Phys. Med. Biol..

[pmbae2230bib14] Dai T (2020). Nanodosimetric quantities and RBE of a clinically relevant carbon-ion beam. Med. Phys..

[pmbae2230bib15] Dokic I (2016). Next generation multi-scale biophysical characterization of high precision cancer particle radiotherapy using clinical proton, helium-, carbon- and oxygen ion beams. Oncotarget.

[pmbae2230bib16] Facchiano S, Ortiz R, Cristoforetti R, D-Kondo N, Jaekel O, Faddegon B, Wahl N (2025). An ion treatment planning framework for inclusion of nanodosimetric ionization detail through cluster dose.

[pmbae2230bib17] Faddegon B (2023). Ionization detail parameters and cluster dose: a mathematical model for selection of nanodosimetric quantities for use in treatment planning in charged particle radiotherapy. Phys. Med. Biol..

[pmbae2230bib18] Faddegon B, Ramos-Méndez J, Schuemann J, McNamara A, Shin J, Perl J, Paganetti H (2020). The TOPAS tool for particle simulation, a Monte Carlo simulation tool for physics, biology and clinical research. Phys. Med..

[pmbae2230bib19] Falk M, Hausmann M (2021). A paradigm revolution or just better resolution—will newly emerging superresolution techniques identify chromatin architecture as a key factor in radiation-induced DNA damage and repair regulation?. Cancers.

[pmbae2230bib20] Fossati P, Matsufuji N, Kamada T, Karger C P (2018). Radiobiological issues in prospective carbon ion therapy trials. Med. Phys..

[pmbae2230bib21] Francis Z, Incerti S, Ivanchenko V, Champion C, Karamitros M, Bernal M A, Bitar Z E (2011). Monte Carlo simulation of energy-deposit clustering for ions of the same LET in liquid water. Phys. Med. Biol..

[pmbae2230bib22] Gardner L L, O’Connor J D, McMahon S J (2024). Benchmarking proton RBE models. Phys. Med. Biol..

[pmbae2230bib23] Garty G, Shchemelinin S, Breskin A, Chechik R, Assaf G, Orion I, Bashkirov V, Schulte R, Grosswendt B (2002). The performance of a novel ion-counting nanodosimeter. Nucl. Instrum. Methods Phys. Res. A.

[pmbae2230bib24] Hagiwara Y, Oike T, Niimi A, Yamauchi M, Sato H, Limsirichaikul S, Held K D, Nakano T, Shibata A (2019). Clustered DNA double-strand break formation and the repair pathway following heavy-ion irradiation. J. Radiat. Res..

[pmbae2230bib25] Harrison N (2024). A novel inverse algorithm to solve IPO-IMPT of proton FLASH therapy with sparse filters. Int. J. Radiat. Oncol. Biol. Phys..

[pmbae2230bib26] Harrison N, Charyyev S, Oancea C, Stanforth A, Gelover E, Zhou S, Dynan W S, Zhang T, Biegalski S, Lin L (2024). Characterizing devices for validation of dose, dose rate, and LET in ultra high dose rate proton irradiations. Med. Phys..

[pmbae2230bib27] Hilgers G, Bug M U, Rabus H (2017). Measurement of track structure parameters of low and medium energy helium and carbon ions in nanometric volumes. Phys. Med. Biol..

[pmbae2230bib28] Incerti S (2010). Comparison of GEANT4 very low energy cross section models with experimental data in water. Med. Phys..

[pmbae2230bib29] Masilela T A M, D-Kondo N, Shin W-G, Ortiz R, Meyer I, LaVerne J A, Faddegon B, Schuemann J, Ramos-Méndez J (2025). TOPAS-nBio-Reg: a regression testing system for track structure simulations in TOPAS-nBio. Phys. Med. Biol..

[pmbae2230bib30] McIntyre M, Wilson P, Gorayski P, Bezak E (2023). A systematic review of LET-guided treatment plan optimisation in proton therapy: identifying the current state and future needs. Cancers.

[pmbae2230bib31] McNamara A, Geng C, Turner R, Mendez J R, Perl J, Held K, Faddegon B, Paganetti H, Schuemann J (2017). Validation of the radiobiology toolkit TOPAS-nBio in simple DNA geometries. Phys. Med..

[pmbae2230bib32] Mietelska M, Pietrzak M, Bancer A, Ruciński A, Szefliński Z, Brzozowska B (2024). Ionization detail parameters for DNA damage evaluation in charged particle radiotherapy: simulation study based on cell survival database. Int. J. Mol. Sci..

[pmbae2230bib33] Nettelbeck H, Rabus H (2011). Nanodosimetry: the missing link between radiobiology and radiation physics?. Radiat. Meas..

[pmbae2230bib34] Nikitaki Z, Pariset E, Sudar D, Costes S V, Georgakilas A G (2020). *In situ* detection of complex DNA damage using microscopy: a rough road ahead. Cancers.

[pmbae2230bib35] Ortiz R, Faddegon B (2024). Creating uniform cluster dose spread‐out Bragg peaks for proton and carbon beams. Med. Phys..

[pmbae2230bib36] Ortiz R, Ramos-Méndez J, Mao J-H, Schulte R, Faddegon B (2025). Evaluation of nanodosimetric quantities for ion radiotherapy treatment planning based on the degree of association of survival with cluster dose. Phys. Med. Biol..

[pmbae2230bib37] Perl J, Shin J, Schumann J, Faddegon B, Paganetti H (2012). TOPAS: an innovative proton Monte Carlo platform for research and clinical applications. Med. Phys..

[pmbae2230bib38] Pszona S, Kula J, Marjanska S (2000). A new method for measuring ion clusters produced by charged particles in nanometre track sections of DNA size. Nucl. Instrum. Methods Phys. Res. A.

[pmbae2230bib39] Rabus H, Ngcezu S A, Braunroth T, Nettelbeck H (2020). “Broadscale” nanodosimetry: nanodosimetric track structure quantities increase at distal edge of spread-out proton Bragg peaks. Radiat. Phys. Chem..

[pmbae2230bib40] Ramos-Méndez J, Burigo L N, Schulte R, Chuang C, Faddegon B (2018). Fast calculation of nanodosimetric quantities in treatment planning of proton and ion therapy. Phys. Med. Biol..

[pmbae2230bib41] Rucinski A, Biernacka A, Schulte R (2021). Applications of nanodosimetry in particle therapy planning and beyond. Phys. Med. Biol..

[pmbae2230bib42] Schuemann J, McNamara A L, Ramos-Méndez J, Perl J, Held K D, Paganetti H, Incerti S, Faddegon B (2018). TOPAS-nBio: an extension to the TOPAS simulation toolkit for cellular and sub-cellular radiobiology. Radiat. Res..

[pmbae2230bib43] Selva A, Colautti P, Conte V (2020). Nanodosimetry of light ions in targets of different size. Radiat. Phys. Chem..

[pmbae2230bib44] Villegas F, Bäckström G, Tilly N, Ahnesjö A (2016). Energy deposition clustering as a functional radiation quality descriptor for modeling relative biological effectiveness: energy deposition clustering for modelling RBE. Med. Phys..

[pmbae2230bib45] Wozny A-S, Rodriguez-Lafrasse C (2023). The ‘stealth-bomber’ paradigm for deciphering the tumour response to carbon-ion irradiation. Br. J. Cancer.

